# Supplementary material for: Seroprevalence and effect of HBV and HCV co-infections on the immuno-virologic responses of adult HIV-infected persons on anti-retroviral therapy
Source: PLoS One. 2022 Nov 23;17(11):e0278037. doi: 10.1371/journal.pone.0278037 (PMC9683579; doi:10.1371/journal.pone.0278037)
Supplement: Attachment — Submitted filename: Response to Reviewers.docx [file pone.0278037.s002.docx]

Medical Laboratory Technology Dept.,

Accra Technical University

P. O. Box GP 156,

Accra, Ghana.

1^st^ October 2022

Dr. Johannes Stortz
Staff Editor
PLOS ONE

Dear Dr. Stortz,

**Re: Resubmission of manuscript reference no. PONE-D-22-19884.**

Please find attached a revised version of our manuscript originally titled “Seroprevalence and effect of HBV and HCV co-infections on the immuno-virologic responses of adult HIV-infected persons on anti-retroviral therapy”, which was submitted for consideration for publication in the PLOS ONE Journal.

We wish to thank you and the reviewers for your insightful comment. These have helped us significantly improve the quality of our manuscript.

In accordance with your comments and that of the Reviewer, revisions to our manuscript have been done using Track Changes in Microsoft Word. The manuscript has been formatted to meet PLOS ONE style requirements, and the authorship list has been amended to include Peter Forson and Sharon Annison. Our point-by-point responses to the Reviewer’s comments are highlighted in yellow. We hope that the revisions in the manuscript and our accompanying responses will be sufficient to make our manuscript suitable for publication in the PLOS ONE Journal.

1. The population size was not provided and important results were not mentioned in the abstract.

Population size has been included accordingly. Results have been included in the Abstract accordingly.

2. The conclusion “Patients with HIV mono-infections are more likely to have sustained HIV viral suppression and higher CD4 cell count, and that HIV/HBV and HIV/HCV coinfections affect the immunologic and virologic response of patients who have initiated highly active antiretroviral therapy” is not supported by results as the associations in table 4 were not statistically significant.

Viral suppression and CD4 cell counts were higher in HIV mono-infected individuals in all instances, howbeit they were not statistically significant. Accordingly, the statement has been amended to include “…though there were no statistical difference”

3. PLHIV not defined at first mention

PLHIV has been defined appropriately

4. The sentence in the introduction about no sero-epidemiology data in HBV and HCV in sub-Saharan Africa need to be qualified. Does it refer to national data? The paper referenced is for a single study not a review paper or a synthesis paper on available data.

The sentence has been amended.

5. Check references eg The tittle for reference 23 has typos.

Error has been corrected.

6. The paper is citing 2010 data for HIV and HBV prevalence in sub-Saharan Africa (22.5 and 52 million) while current epidemiology data is available for the 2 pathogens.

Current data (2021 for HIV and 2019 for HBV) have been added, and old data deleted.

7. HBsAg was defined several times in the paper

The repeated definition has been deleted.

8. Manufactures details were not provided for the Advanced Quality One Step Tests.

Manufacturer’s details have been added accrodingly.

9. Include confidence intervals for prevalence rates and just report the prevalence for the reactive results. The non reactive results are implied. Similar to male or female.

Implied results have been deleted accordingly, and confidence intervals have been added to our study’s prevalence.

10. For statistical analysis section include the p value significance cut off.

P value cut-off has been included.

11. Reference of WHO guidelines used for HIV RNA categories was not provided

Reference has been provided.

12. Include percentages here “Patients with viral load below undetectable levels were made up of 177/266 females and 89/266 males”.

Percentages have been added appropriately.

13. Were there any HBV/HIV or HIV/HCV participants with virologic failure?

Results for HBV/HIV and HCV/HIV viral suppression can be found in Paragraph 2 line 20 under Results of **Comparison of patients’ demography and clinical characteristics,** and it has been highlighted in yellow for easy verification. It is also shown in Table 4.

14. Table 7 referred here might be a typo “……………detectable in 28 (66.7%) and undetectable in 14 (33.3%) (Table 7)”

It is a typographical error and has been corrected. It is Table 4.

15. The prevalence rates should also be compared to previous studies from Ghana

Previous studies in Ghana has been compared with current study.

16. Isn’t this “There was a higher proportion (64.3%) of female HIV/HBV co-infected patients than males (35.7%)” because there were more females in the study in general? Eg There were higher proportions of females (66.6%) than males (33.4%)

That is the case. There were more female participants than males, and that has been stated accordingly. In addition, other reasons that may have accounted for the gender disparity have also been stated.

17. 17. In the discussion it is stated that “in this study the mean HIV viral load of female participants was slightly higher but the difference was not statistically significant. The sentence in the discussion about HIV RNA appearing to be higher is misleading in the discussion is the difference was not statistically different

The statement has been amended for clarity.

18. The study concluded that HIV/HBV coinfection was low but 8.4% is not necessarily low. Also check the interpretation of non significant results in the conclusion. Which HCV treatment would be included as part of the ART regimen in reference to this sentence “The study therefore recommends the use of ARVs active against HBV and HCV in ART regimens for HIV co-infected patients regardless of the CD4 cell count level”. Also considering the fact that there was only one patient with HCV in this study.

The authors agree with the Reviewer that 8.4% is not necessarily low. We generalized the HIV/HBV and HIV/HCV together due to the low (0.2%) prevalence of HIV/HCV. The sentence has been deleted accordingly. Approved ARVs active against HBV have been added.

Thank you for your consideration. We look forward to hearing from you.

Sincerely,

Lawrence Annison

(lannison@atu.edu.gh)
