# Supplementary material for: Seroprevalence and effect of HBV and HCV co-infections on the immuno-virologic responses of adult HIV-infected persons on anti-retroviral therapy
Source: PLoS One. 2022 Nov 23;17(11):e0278037. doi: 10.1371/journal.pone.0278037 (PMC9683579; doi:10.1371/journal.pone.0278037)
Supplement: Attachment — Submitted filename: Response to Reviewers.docx [file pone.0278037.s003.docx]

Medical Laboratory Technology Dept.,

Accra Technical University

P. O. Box GP 156,

Accra, Ghana.

4^th^ November, 2022

Dr. Yuri Khudyakov

Academic Editor
PLOS ONE

Dear Dr. Khudyakov,

**Re: Resubmission of manuscript reference no. PONE-D-22-19884R1.**

Please find attached a revised version of our manuscript originally titled “Seroprevalence and effect of HBV and HCV co-infections on the immuno-virologic responses of adult HIV-infected persons on anti-retroviral therapy”, which was submitted for consideration for publication in the PLOS ONE Journal.

We wish to thank you and the reviewers for your insightful comment. These have helped us significantly improve the quality of our manuscript.

In accordance with your comments and that of the Reviewer, revisions to our manuscript have been done using Track Changes in Microsoft Word. The Reviewer raised concerns about the conclusions not being supported by the data provided. Our response to the Reviewer’s comment in point 6 is highlighted in yellow. We hope that the revisions in the manuscript and our accompanying response will be sufficient to make our manuscript suitable for publication in the PLOS ONE Journal.

Academic Editor’s comments:

Your revised manuscript was reviewed by one expert in the field. The reviewer indicated that one of the comments was not satisfactorily addressed and your conclusions are still not supported by the data. Please respond properly to this comment.

**Comments** **to the Author**

1. If the authors have adequately addressed your comments raised in a previous round of review and you feel that this manuscript is now acceptable for publication, you may indicate that here to bypass the “Comments to the Author” section, enter your conflict of interest statement in the “Confidential to Editor” section, and submit your "Accept" recommendation.

Reviewer #1: (No Response)

2. Is the manuscript technically sound, and do the data support the conclusions?

Reviewer #1: Partly

3. Has the statistical analysis been performed appropriately and rigorously?

Reviewer #1: I Don't Know

4. Have the authors made all data underlying the findings in their manuscript fully available?

Reviewer #1: Yes

5. Is the manuscript presented in an intelligible fashion and written in standard English?

Reviewer #1: Yes

6. Review Comments to the Author

Reviewer #1: Comment number 2 is still not addressed. The conclusion “Patients with HIV mono-infections are more likely to have sustained HIV viral suppression and higher CD4 cell count, and that HIV/HBV and HIV/HCV coinfections affect the immunologic and virologic response of patients who have initiated highly active antiretroviral therapy” is not supported by results as the associations in table 4 were not statistically significant.

The authors agree with the Reviewer that associations in Table 4 were not statistically significant. Conclusions have been revised accordingly in the Abstract and Conclusion sessions to reflect the data as captured in Table 4. The revision can be found in the Track Changes attached.

Thank you for your consideration. We look forward to hearing from you.

Sincerely,

Lawrence Annison

(lannison@atu.edu.gh)
